# Diagrammatic Simplification of Linearized Coupled Cluster Theory

**DOI:** 10.1021/acs.jpca.5c03203

**Published:** 2025-06-26

**Authors:** Kevin Carter-Fenk

**Affiliations:** Department of Chemistry, 6614 University of Pittsburgh, Pittsburgh, Pennsylvania 15218, United States

## Abstract

Linearized Coupled Cluster Doubles (LinCCD) often provides near-singular energies in small-gap systems that exhibit static correlation. This has been attributed to the lack of quadratic *T̂*_2_^2^ terms that typically balance out small energy denominators in the CCD amplitude equations. Herein, I show that exchange contributions to ring and crossed-ring contractions (not small denominators *per se*) cause the divergent behavior of LinCC­(S)­D approaches. Rather than omitting exchange terms, I recommend a regular and size-consistent method that retains only linear ladder diagrams. As LinCCD and configuration interaction doubles (CID) equations are isomorphic, this also implies that simplification (rather than quadratic extensions) of CID amplitude equations can lead to a size-consistent theory. Linearized ladder CCD (LinLCCD) is robust in statically correlated systems and can be made $O\left(n_{\mathrm{occ}}^{4}n_{\mathrm{vir}}^{2}\right)$ with a hole–hole approximation. The results presented here show that LinLCCD and its hole–hole approximation can accurately capture energy differences, even outperforming full CCD and CCSD for noncovalent interactions in small-to-medium sized molecules, setting the stage for further adaptations of these approaches that incorporate more dynamical correlation.

## Graphical abstract

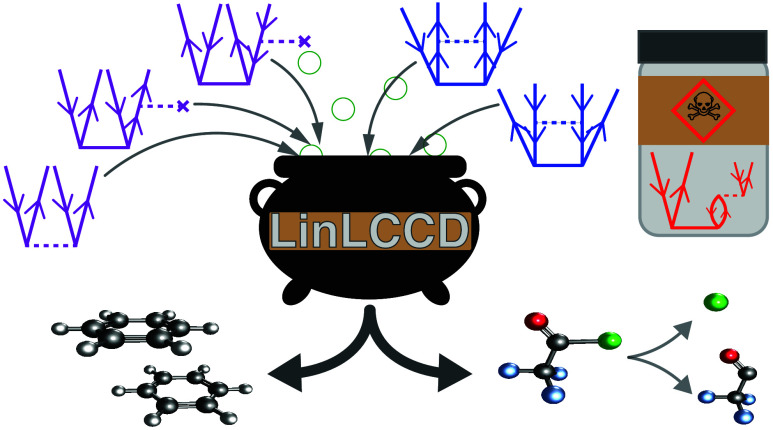

## 1 Introduction

Coupled cluster (CC) theory with double substitutions (CCD) is the simplest form of CC that captures electron correlation.^1^ There are a host of advantages to linearized CCD methods (LinCCD) over full CCD,^2,3^ including reductions in memory demands, ease of spin-adapting the LinCCD wave function (albeit there is no rigorous wave function in linearized CC approximations),^4^ and simpler physical interpretation of the equations. My research group is particularly interested in the Hermitian formulation that is offered by linearized CC methods, as this can be useful in developing excited-state theories.^5^ Hermitian approaches are also quite powerful in the sense that they satisfy the generalized Hellmann–Feynman theorem, permitting simpler evaluation of forces (whereas left-eigenvectors are required in non-Hermitian CC approaches).^6^

However, linearized CC approaches often encounter near singularities in small-gap systems, affecting the performance of LinCCD away from equilibrium.^7^ Small orbital-energy gaps are often a qualitative indicator of static correlation,^8^ where the near-singular behavior of LinCCD can be further understood as a deficiency resulting from the lack of quadratic *T̂*_2_^2^ terms that fold in higher-order correlation effects necessary to describe static correlation. In other words, LinCCD lacks the implicit account for quadruple excitations that is found in the CCD amplitude equations, making it unable to counteract small energy denominators. Consequently, LinCCSD has been combined with Tikhonov regularization^9^ as a means of sidestepping divergences.^7^ Multireference LinCC approaches have also been developed to avoid divergences in systems that exhibit static correlation, albeit at increased cost.^10–12^ Furthermore, LinCC methods have been applied to capture additional correlation effects atop geminal reference states that naturally incorporate static correlation.^13–16^

Beyond LinCCD, there are classes of CC approaches that attempt to correct errors within single-reference CC that arise due to static correlation. These “addition-by-subtraction” (ABS) CC methods take the seemingly paradoxical approach of removing components of the *T̂* operator that are found to be particularly ill-behaved in the face of static correlation.^17^ Perhaps the most well-known ABS-CC approach is pair CCD,^18^ where only pair double substitution clusters are retained, leading to an approach that can describe single and double-bond dissociation.^19–22^ Alternatively, it is also possible to decouple the singlet- and triplet-paired amplitudes in CCD to achieve similarly well-behaved bond dissociation curves.^23,24^ Though, such singlet/triplet-pair couplings occur through a quadratic term in the CCD equations so the divergence of LinCCD must be attributable to other factors.

Another flavor of ABS-CC approach restricts the CCD equations to certain classes of diagrams. For instance, CCD with only ring diagrams is equivalent to the particle-hole random-phase approximation (ph-RPA)^25–27^ and is especially applicable in the case of the high-density homogeneous electron gas. Beyond single-reference approaches, diagrammatic resummations of the ring diagrams have been recently applied to extend ph-RPA to the multireference case.^28^

At the other end of the spectrum, CCD restricted to ladder diagrams (ladder-CCD) is formally equivalent to particle–particle RPA (pp-RPA)^29,30^ and is especially suitable in the limit of the low-density electron gas due to its explicit account of particle–particle correlations. Diagrammatic analysis of the ring and ladder CC equations has revealed that ring and ladder diagrams mainly describe long and short-ranged correlation effects, respectively.^31^ These naturally imposed length scales have been leveraged in combinations of ladder- and ring-CCD via range-separation techniques, leading to promising methods for describing systems that do not fall into either extreme.^32^

In this work, I present an ABS linearized CCD approach that linearizes the ladder CCD amplitude equations. By removing the terms associated with ring and crossed-ring diagrams from LinCCD, the resultant linearized ladder CCD (LinLCCD) approach avoids the near-singularities encountered in these diagrams. Furthermore, the isomorphism between LinCCD and configuration interaction with double substitutions (CID) suggests that LinCCD equations are not size-consistent.^1^ A lack of size-consistency implies that, for well-separated molecular fragments *A* and *B*, *E*_*A*∪*B*_ = *E*_*A*_ + *E*_*B*_ is not satisfied by LinCCD/CID. While quadratic corrections have been added to CID to obtain CCD equations,^33–38^ revealing the role of *T̂*_2_^2^ terms in size-consistent approaches, I propose removing ring/crossed-ring terms from the LinCCD (or equivalently CID) equations as an alternative route to obtain a size-consistent, size-extensive, orbital invariant, and naturally regular method.

## 2 Theory

Throughout this work, I will denote occupied orbitals as *i*, *j*, *k*, *l*, ···, virtual orbitals as *a*, *b*, *c*, *d*, ···, and general unspecified orbitals as *p*, *q*, *r*, *s*, ···. The abbreviations *n*_*v*_ and *n*_*o*_ will be used for the number of virtual orbitals and the number of occupied orbitals, respectively. Einstein summation notation is used except in limited cases where the summation is explicitly written out.

### 2.1 Linearized Coupled Cluster Theory

LinCCD invokes the approximation that the usual exponential parameterization of the wave function

1 $$\left|\Psi_{\mathrm{CC}}\right\rangle =e^{\mathrm{T̂}}\left|\Phi_{\mathrm{HF}}\right\rangle$$

is Taylor-expanded through first order such that

2 $$\left|\Psi_{\mathrm{LinCC}}\right\rangle \approx \left(1+\mathrm{T̂}\right)\left|\Phi_{\mathrm{HF}}\right\rangle$$

By retaining only strongly connected diagrams, the LinCCD energy can be cast in terms of a Hermitian Hamiltonian

3 $$E=\left\langle \Phi_{\mathrm{HF}}\right|{\left[\left(1+{\mathrm{T̂}}_{2}^{†}\right)Ĥ\left(1+{\mathrm{T̂}}_{2}\right)\right]}_{\mathrm{SC}}\left|\Phi_{\mathrm{HF}}\right\rangle$$

where

4 $${\mathrm{T̂}}_{2}=\sum_{\begin{array}{l} i>j\\ a>b \end{array}}t_{\mathrm{ij}}^{\mathrm{ab}}{â}_{a}^{†}{â}_{i}{â}_{b}^{†}{â}_{j}$$

is the usual double-substitution operator and the subscript SC indicates that only the strongly connected diagrams are retained. Connected diagrams are defined as those whose components are all connected via directed lines. Strongly connected diagrams are a subclass of connected diagrams in which – for operators with dual-space components such as *T̂* ^†^ *ĤT̂* in eq 3 – the removal of one *T̂* ^†^ or *T̂* still results in a connected diagram. On the other hand, if such a removal results in a disconnected diagram the term “weakly connected” is used.^39,40^ Restricting Hermitian linearized CC equations to the subclass of strongly connected diagrams ensures size-extensivity.

The LinCCD doubles amplitude equations are

0=vijab−Pij(tkjabfik)+Pab(fcatijcb)+12tklabvijkl+12vcdabtijcd+PijPab(vicaktkjcb) 5

where *v*_*pq*_^*rs*^ are antisymmetrized 2-electron integrals ⟨*rs*∥*pq*⟩ and $P_{\mathrm{pq}}=1-p↔q$ are index permutation operators. The first 3 terms (first line) of eq 5 are often referred to as the *driver* terms (the latter two of which are responsible for the energy denominator in perturbation theory). The fourth and fifth terms (middle line of eq 5) are associated with *ladder* diagrams, and the final term (last line) emerges from *ring* and *crossed-ring* diagrams. The LinCCD diagrams are explicitly shown alongside their corresponding mathematical incarnation in Figure 1. For more detail pertaining to the CCD (and LinCCD) diagrams (including the basis set convergence of each term) the reader is referred to ref 41. Finally, the LinCCD energy expression in the spatial-orbital basis is explicitly:

6 $$E=\frac{1}{4}v_{\mathrm{ab}}^{\mathrm{ij}}t_{\mathrm{ij}}^{\mathrm{ab}}$$

**Figure 1:**
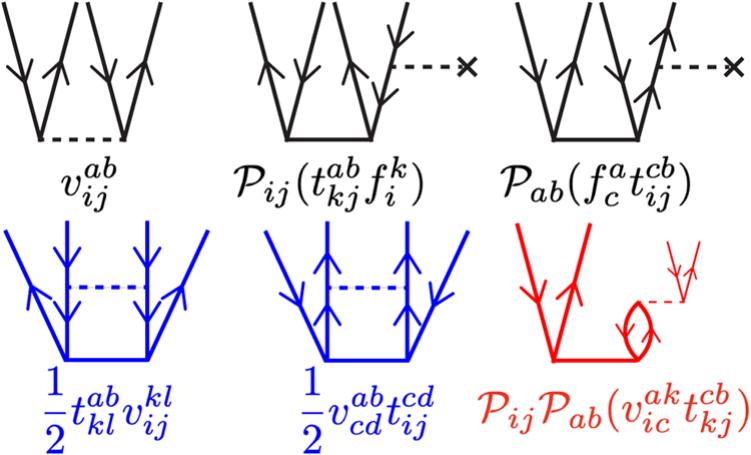
Diagrammatic representation of the linearized CCD equations. The “driver” terms are displayed in black, “ladder” terms in blue, and “ring and crossed-ring” in red. When permutation operations are shown, the associated diagram corresponds to the contraction displayed in parentheses.

### 2.2 Linearized Ladder Coupled Cluster Doubles

Interestingly, to my knowledge it has yet to be observed that retaining only *driver* terms and *ladder*-type diagrams within LinCCD leads to naturally regular equations that are strongly resistant to divergence in molecular systems. Notably, linearized ladder approximations in various combinations with other RPA terms have been explored in the context of the homogeneous electron gas,^42–46^ but – to the best of my knowledge – were essentially abandoned before being applied in the context of chemistry. Given the tremendous volume of interest in pp-RPA/ladder-CCD approaches in quantum chemistry^47–54^ it appears pertinent to explore linearized ladder approximations.

Applying the same precedent of diagrammatic simplification as pp-RPA/ladder-CCD, I restrict LinCCD to ladder diagrams yielding

7 $$0=v_{\mathrm{ij}}^{\mathrm{ab}}+\left(f_{c}^{a}\delta_{d}^{b}+\delta_{c}^{a}f_{d}^{b}\right)t_{\mathrm{ij}}^{\mathrm{cd}}-\left(f_{i}^{k}\delta_{j}^{l}+\delta_{i}^{k}f_{j}^{l}\right)t_{\mathrm{kl}}^{\mathrm{ab}}+\frac{1}{2}t_{\mathrm{kl}}^{\mathrm{ab}}v_{\mathrm{ij}}^{\mathrm{kl}}+\frac{1}{2}v_{\mathrm{cd}}^{\mathrm{ab}}t_{\mathrm{ij}}^{\mathrm{cd}}$$

These LinLCCD equations are naturally regular. In principle, the inclusion of only ladder diagrams incorporates the most important contributions for describing strong correlation. However, to understand precisely how this is the case, it is helpful to notice that the contractions in terms 2 and 5 and terms 3 and 4 can be grouped together

8 $$\left(f_{c}^{a}\delta_{d}^{b}+\delta_{c}^{a}f_{d}^{b}+\frac{1}{2}v_{\mathrm{cd}}^{\mathrm{ab}}\right)t_{\mathrm{ij}}^{\mathrm{cd}}-\left(f_{i}^{k}\delta_{j}^{l}+\delta_{i}^{k}f_{j}^{l}-\frac{1}{2}v_{\mathrm{ij}}^{\mathrm{kl}}\right)t_{\mathrm{kl}}^{\mathrm{ab}}=-v_{\mathrm{ij}}^{\mathrm{ab}}$$

By choosing a clever basis for eq 8, such as the one that diagonalizes the *n*_*v*_^2^ × *n*_*v*_^2^ matrix in term 1 and the *n*_*o*_^2^ × *n*_*o*_^2^ matrix in term 2, the amplitude equation can be reduced to a highly revealing linear form. Specifically, one can define a particle–particle (pp)- and hole–hole (hh)-blocked super Fock matrix with elements

9a $$F_{\mathrm{cd}}^{\mathrm{ab}}=f_{c}^{a}\delta_{d}^{b}+\delta_{c}^{a}f_{d}^{b}$$

9b $$F_{\mathrm{ij}}^{\mathrm{kl}}=f_{i}^{k}\delta_{j}^{l}+\delta_{i}^{k}f_{j}^{l}$$

where the eigenvalues of eq 9, the pp-Fock matrix (**F**_pp_), and eq 10, the hh-Fock matrix (**F**_hh_), are the hh- and pp-pair energies as estimated by sums of canonical one-particle orbital energies. Taking **V**_pp_ and **V**_hh_ as matrix representations of the pp- and hh-integrals from eq 8, one can solve the eigenvalue problems

10a $$\left(F_{\mathrm{pp}}+\frac{1}{2}V_{\mathrm{pp}}\right)X=\lambda X$$

10b $$\left(F_{\mathrm{hh}}-\frac{1}{2}V_{\mathrm{hh}}\right)Y=\eta Y$$

where the eigenvalues are pp-pair (**λ**) and hh-pair (**η**) orbital energies in the dressed orbital basis, respectively. They can be decomposed into a one-particle pair contribution and a two-particle “dressing” supplied by the integrals

11a $$\lambda_{\mathrm{ab}}={\mathrm{F̃}}_{\mathrm{ab}}+\frac{1}{2}{ṽ}_{\mathrm{ab}}^{\mathrm{ab}}$$

11b $$\eta_{\mathrm{ij}}={\mathrm{F̃}}_{\mathrm{ij}}-\frac{1}{2}{ṽ}_{\mathrm{ij}}^{\mathrm{ij}}$$

where ${\mathrm{F̃}}_{\mathrm{pq}}$ are the contributions from one-particle pair energies and the tilde designates quantities that are in the dressed orbital basis.

Rotation of the supermatrices defined above by eigenvectors **X** in the pp space and **Y** in the hh space leads to a diagonal representation of eq 8

12 $$\left(\lambda_{\mathrm{ab}}-\eta_{\mathrm{ij}}\right){\mathrm{t̃}}_{\mathrm{ij}}^{\mathrm{ab}}=-{ṽ}_{\mathrm{ij}}^{\mathrm{ab}}$$

The quantity in parentheses is diagonal and is thus invertible, leading to the amplitudes

13 $${\mathrm{t̃}}_{\mathrm{ij}}^{\mathrm{ab}}=-\frac{{ṽ}_{\mathrm{ij}}^{\mathrm{ab}}}{\left({\mathrm{F̃}}_{\mathrm{ab}}+\frac{1}{2}{ṽ}_{\mathrm{ab}}^{\mathrm{ab}}\right)-\left({\mathrm{F̃}}_{\mathrm{ij}}-\frac{1}{2}{ṽ}_{\mathrm{ij}}^{\mathrm{ij}}\right)}$$

Here, the elements of **λ** and **η** are expanded to emphasize that the denominator is constructed of inseparable dressed pair energies. This distinguishes LinLCCD as a coupled pair theory as opposed to the independent electron pair theory that constitutes second-order Møller–Plesset perturbation theory (MP2), to which these equations bear a striking resemblance. Finally, note that this basis transformation was accomplished via independent occupied-occupied and virtual–virtual orbital rotations, so the orbital-invariant LinLCCD energy does not change.

Such an isomorphism between MP2 and CCD equations has been noticed before, but only in the context of mosaic CCD.^55^ In the linearized ladder context, the hh- and pp-pair energies have been shifted by the hh and pp integrals, respectively. This essentially results in a set of screened first-order amplitudes wherein the energy gap is widened by adding hh correlations to the one-particle occupied pair energies and pp correlations to one-particle virtual pair energies, making LinLCCD robust against divergence in small-gap systems. Unlike mosaic CCD and myriad other renormalized MP2 theories,^56–60^ the LinLCCD gap is widened in an amplitude-independent way, suggesting that the LinLCCD equations could be solved noniteratively, albeit such an approach would be quite impractical as it would require the diagonalization of a *n*_*v*_^2^ × *n*_*v*_^2^ matrix.

Incredibly, removing the ring and crossed-ring contractions from LinCCD also corrects the size-consistency errors in the parent method. I note that beyond size-consistency, LinLCCD is also size-extensive and orbital invariant. As the LinLCCD equations can be recast in the form of eq 16, the proof for size-consistency in this basis is trivial. Consider a system wherein molecules *A* and *B* are sufficiently far apart that their respective molecular orbitals may be trivially fragment-ascribed. After a rotation into the aforementioned dressed orbital basis eq 16 becomes zero for all sums over disjoint orbitals by means of the electron repulsion integrals in the numerator, ensuring that the resultant energy satisfies *E*_*A*∪*B*_ = *E*_*A*_ + *E*_*B*_.

My group is particularly interested in low-scaling approximations that describe static correlation qualitatively. While I will demonstrate the utility of LinLCCD, it does retain the most expensive $O\left(n_{o}^{2}n_{v}^{4}\right)$ particle–particle ladder term that is responsible for the $O\left(N^{6}\right)$ cost of CCD. In the spirit of exploring low-scaling variants of linear ladder theories, I introduce one further approximation by completely removing the costly particle–particle ladder term to achieve

14 $$\left(f_{c}^{a}\delta_{d}^{b}+\delta_{c}^{a}f_{d}^{b}\right)t_{\mathrm{ij}}^{\mathrm{cd}}-\left(f_{i}^{k}\delta_{j}^{l}+\delta_{i}^{k}f_{j}^{l}-\frac{1}{2}v_{\mathrm{ij}}^{\mathrm{kl}}\right)t_{\mathrm{kl}}^{\mathrm{ab}}=-v_{\mathrm{ij}}^{\mathrm{ab}}$$

thus shifting only the occupied-pair energies by the hole–hole ladder term. Unlike the LinLCCD case, there is no diagrammatic justification for removing the particle–particle ladder term, but the resultant LinLCCD­(hh) approach is a potentially fruitful approximation that scales much more favorably as $O\left(n_{o}^{4}n_{v}^{2}\right)$ .

It should be noted that the LinLCCD­(hh) approximation does not sacrifice orbital invariance, size-consistency, or size-extensivity. Such claims can be verified by applying analogous occupied-occupied orbital rotations to eq 17 such that term 2 becomes diagonal. Once again, this gives way to a set of dressed amplitude equations that can be written as

15 $$\left(\varepsilon_{a}+\varepsilon_{b}-\eta_{\mathrm{ij}}\right){\mathrm{t̃}}_{\mathrm{ij}}^{\mathrm{ab}}=-{ṽ}_{\mathrm{ij}}^{\mathrm{ab}}$$

where now only the occupied pair orbital energies have been dressed with hh correlation. While these basis transformations are revealing as to the underlying nature of various linearized CC approximations, in practice my implementation solves eq 8 self-consistently.

## 3 Results and Discussion

### 3.1 Bond Breaking

I first show the relative robustness of LinLCCD methods through a few simple bond dissociation potential energy surfaces. The simplest case of H_2_ in the STO-3G basis is shown in Figure 2a. As expected, LinCCD diverges rapidly as the H–H bond is stretched. However, both LinLCCD and the further pruned LinLCCD­(hh) approaches smoothly dissociate H_2_ toward some limit, which is exact in the case of LinLCCD­(hh). Of course, I note that these results also show that LinLCCD is no longer exact for all two electron systems, but neither is its (chemically very useful) parent ladder approximation, pp-RPA.

**Figure 2:**
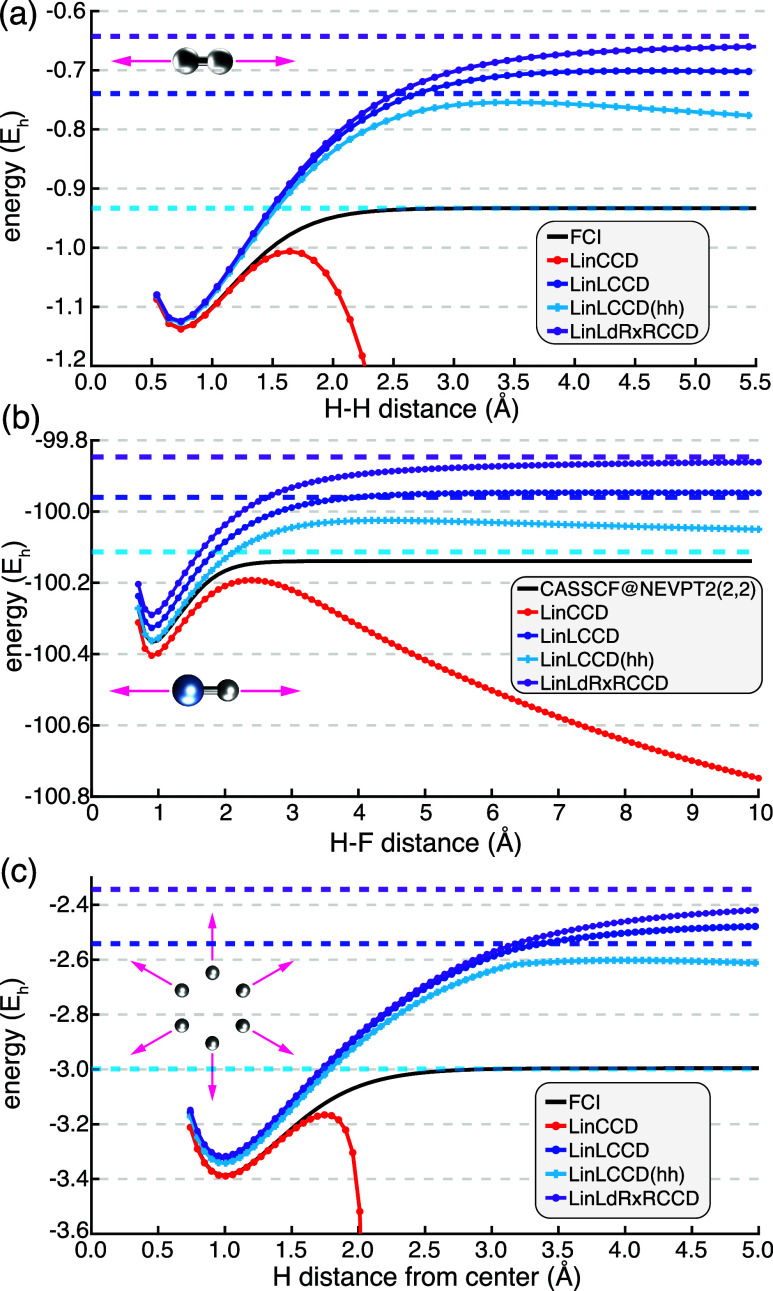
Dissociation curves for (a) hydrogen molecule in the minimal STO-3G basis set,^61^ (b) Hydrogen fluoride molecule in the aug-cc-pVQZ basis,^62,63^ and (c) H_6_ in the cc-pVDZ basis. Dashed lines of like-color indicate the dissociation limit for a particular method. Dissociation limits were estimated at *R* = 10^6^ Å, except in the case of H_6_ where *R* = 10^3^ Å was used instead due to convergence difficulties.

Interestingly, if the linear direct ring and crossed-ring terms (i.e., without antisymmetrizing the two-electron repulsion integrals) are retained alongside the fully antisymmetric ladder terms, the resultant LinLdRxRCCD approach also does not diverge. LinLdRxRCCD differs from full LinCCD only by the exchange component of the linear ring and crossed-ring contractions, implying that the term most responsible for the instability of LinCCD is not the small orbital-energy denominator *per se*, but the exchange ring/crossed-ring terms. In the case of HF molecule dissociation in Figure 2b, this finding helps to explain why regularization by means of eliminating near-zero denominator terms in LinCCSD is only somewhat effective,^7^ eventually breaking down at large *R*_H–F_, whereas LinLCCD, LinLCCD­(hh), and LinLdRxRCCD are all stable out to 10^6^ Å.

I note that others have put forth that the divergence of CC equations alongside other deficiencies in statically correlated systems manifest due to various exchange terms.^25,30,64,65^ Similar instabilities were also recently reported for renormalized propagator methods that include ring and crossed-ring exchange terms.^66^ Of course, removing exchange terms can lead to undesirable self-interaction artifacts, as is well-known in the case of direct ring CCD (otherwise known as the direct ph-RPA), which overbinds significantly at equilibrium geometries.^67–70^ Though less common, removing exchange terms from ladder CCD has also been explored but appears to lead to less satisfactory results for bond dissociation energies.^71^ Thus, if a self-interaction-free theory is desired that can smoothly dissociate bonds to a clear asymptotic limit, LinLCCD and LinLCCD­(hh) represent suitable options.

Lastly in the series of bond dissociation curves, I investigate hexagonal H_6_ dissociation in Figure 2c. The hexagonal H_6_ system is prototypical of strongly correlated systems in chemistry and is reminiscent of the Hubbard model Hamiltonian. As the H_6_ ring is expanded LinCCD rapidly diverges while all methods that exclude ring/crossed-ring exchange diagrams remain stable. The estimated asymptotic limit of LinLCCD­(hh) appears somewhat deceptive as plotted because it very slightly overestimates the correlation energy at large *R*, dipping below the full configuration interaction (FCI) reference curve by about 4 mE_h_. While the LinLCCD­(hh) potential curve is far too repulsive at intermediate *R*, the asymptotic limit remains very impressive for such an approximate scheme. Overall, all methods that remove the exchange ring/crossed-ring terms are robust for the bond stretching coordinates investigated in Figure 2 while LinCCD fails in all cases, suggesting that ring/crossed-ring exchange contractions are to blame for the near-singular behavior of LinCCD.

### 3.2 Non-Bonded Interactions

Recall that LinLCCD is an approximation to pp-RPA, so it should not be expected to be a quantitatively accurate approach for total energies as pp-RPA—and therefore LinLCCD—does not offer ideal coverage of dynamical correlation effects.^29,47,55^ These deficiencies can be accounted for in pp-RPA by combination with density functionals, but I will not explore this here.^49,51,72–75^ The lack of dynamical correlation can be immediately seen in the potential energy curves of Figure 2, as LinLCCD and LinLCCD­(hh) both underestimate the magnitude of the correlation energy relative to FCI and LinCCD near equilibrium. However, much of the utility in RPA methods comes from how accurately they predict energy differences rather than the total energies themselves.^29^ In this section I will explore the accuracy of LinLCCD for noncovalent interactions computed via

16 $$\Delta E_{\mathrm{int}}=E_{\mathrm{AB}}-E_{A}-E_{B}$$

where *E*_*AB*_ is the energy of the complex and *E*_*X*_ is the monomer energy of fragment *X*. All interaction energy calculations have been counterpoise corrected.^76^

First, I examine the performance of LinLCCD, LinLCCD­(hh), LinCCD, CCD, and CCSD on the A24 data set of small dimers in Figure 3a.^77^ These results are largely unremarkable as all methods perform statistically about the same with relatively low errors ranging between 0.1–0.2 kcal/mol. Some notable differences are seen in Figure 3b for the S22 data set which features several medium-sized π-stacked systems.^78,79^ Interestingly, while LinLCCD and LinLCCD­(hh) perform similarly with quite low mean absolute errors (MAE) of 0.4 kcal/mol, LinCCD performs poorly with a MAE of 1.4 kcal/mol. This poorer performance is in line with the full CCD and CCSD methods, which also under-perform on S22. While it is sensible that LinCCD, CCD, and CCSD perform similarly at equilibrium geometries, it is reasonable to wonder whether any particular interaction type (H-bonding, dispersion-bound, π-stacking, or mixed interactions) is responsible for the uniformly poor performance of these methods. However, Figure S1 suggests that the performance of all approaches remains consistent across interaction types. This is especially interesting in the case of LinLCCD methods, which are akin to renormalized MP2. Whereas MP2 tends to have a significant propensity to overbind π-stacked complexes by upward of 100%,^80,81^ no such bias is noted in LinLCCD approaches for the dimers in S22.

**Figure 3:**
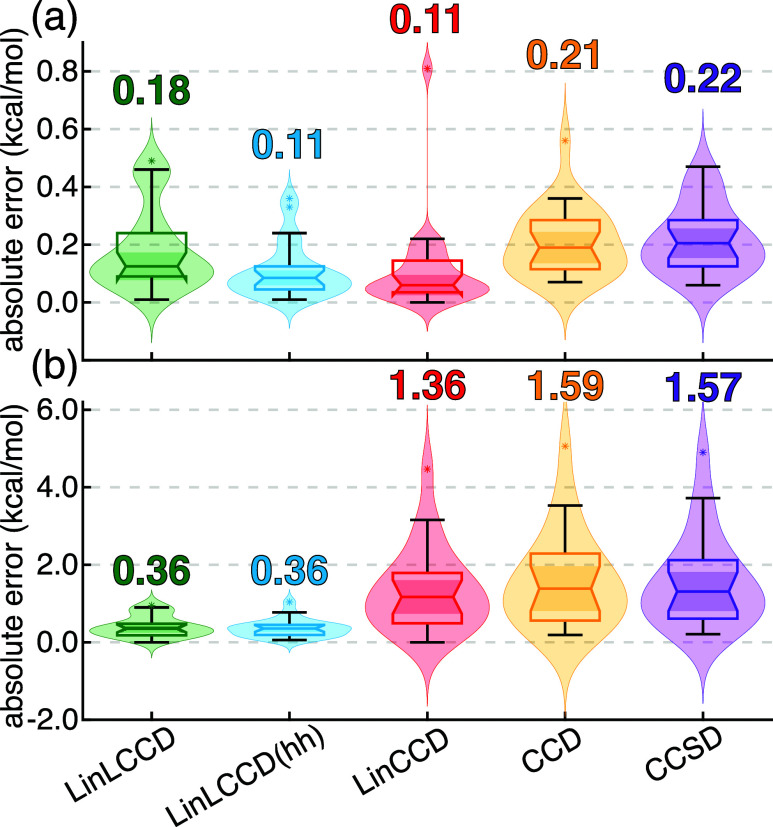
Error statistics for noncovalent interaction energies extrapolated to the complete basis set limit for (a) the A24 set of small dimers and (b) the S22 data set of small to medium sized dimers. The inset numbers indicate the mean absolute error in kcal/mol.

Both LinLCCD and LinLCCD­(hh) methods appear to perform at least as well as regularized perturbation theory approaches.^82,83^ These results suggest that, despite a lack of dynamical correlation, the energy differences obtained with LinLCCD approaches are quite accurate for nonbonded interactions, surpassing those of full CCD and CCSD.

In an effort to emphasize the relative affordability of the $O\left(n_{o}^{4}n_{v}^{2}\right)$ LinLCCD­(hh) approximation, I also present complete basis set limit extrapolated interaction energies for the L7 data set of large dimers in Figure 4.^85^ I compare to the complete basis set limit domain-localized pair natural orbital (DLPNO)^86–88^ CCSD­(T_0_) benchmark data of Lao and co-workers.^84^ I note that DLPNO–CCSD­(T_0_) results are quite sensitive to the particular thresholds chosen, so the benchmark data have some unknown error in addition to those imposed by the (*T*_0_) correction. This error is roughly 2–6 kcal/mol for the π-stacked complexes in L7.^89^

**Figure 4:**
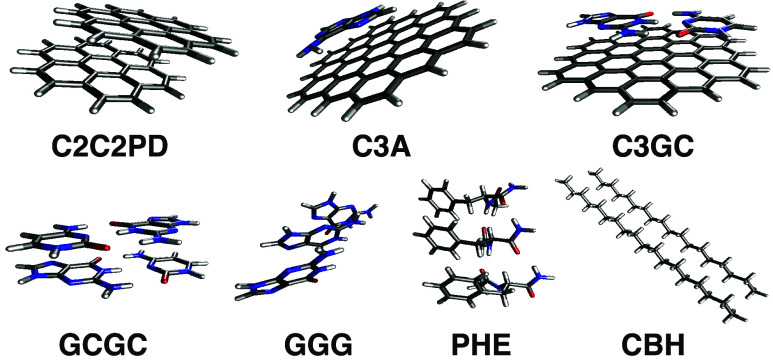
Systems that comprise the L7 data set along with their commonly employed acronyms.

The results in Table 1 show that MP2 dramatically overestimates the interaction energies in L7,^81^ which features several large π-stacked systems. As LinLCCD­(hh) can be viewed as a renormalized MP2, it is of interest to contrast its performance with MP2 and with the renormalized MP2 approach known as size-consistent Brillouin–Wigner perturbation theory (BW-s2).^83^ The results in Table 1 suggest that including hole–hole relaxation in the one-particle energies can temper the overestimated interaction energies of conventional MP2, but not dramatically so. The MAE is reduced relative to MP2 by nearly 2 kcal/mol, which is an improvement but suggests that linear ladder correlation is insufficient to achieve quantitative accuracy. The renormalization supplied by BW-s2 is somewhat more effective at suppressing overcorrelation in the largest π-stacked systems than LinLCCD­(hh), but less aggressive in systems like GCGC, GGG, and CBH, leading to an overall MAE that is about 0.6 kcal/mol lower than LinLCCD­(hh). That said, the results between BW-s2 and LinLCCD­(hh) are comparable to within 12% of one another. While I have shown that LinLCCD­(hh) is affordable enough to be applied to such large systems and that the results are somewhat improved relative to MP2, some empiricism,^59^ or other means of incorporating additional many-body screening effects could beget improvements.

**Table 1:** Interaction Energies for L7 Data Set (kcal/mol)

system	LinLCCD(hh)^a^	BW-s2^a^,^b^	MP2^a^,^b^	CCSD(T_0_)^a^,^c^
C2C2PD	–34.54	–33.32	–38.08	–20.93 ± 0.44
C3A	–25.06	–24.11	–27.09	–17.49 ± 0.31
C3GC	–41.54	–40.40	–45.37	–29.24 ± 0.91
GCGC	–16.50	–17.00	–18.99	–13.54 ± 0.27
GGG	–3.31	–3.63	–4.54	–2.08 ± 0.09
CBH	–9.40	–10.90	–11.83	–11.00 ± 0.17
PHE	–25.94	–25.73	–26.32	–25.46 ± 0.01
MAE	5.36	4.78	7.18	

a Extrapolated to CBS limit.

b From ref 59.

c DLPNO–CCSD­(T_0_) from ref 84.

### 3.3 Quest #8 Singlet/Triplet Gaps

Spin state energetics of transition metal complexes are a key quantity in the predictive modeling of energy relevant systems such as photoredox catalysts that are used to generate solar fuels such as H_2_ and to facilitate organic syntheses.^90–93^ While there remains a paucity of benchmark-quality spin-state energetics data for transition metal complexes in the literature, the small Quest #8 data set of Loos and co-workers does provide excellent data for comparisons with nonrelativistic quantum chemistry methods.^94,95^ The Quest #8 set contains 11 diatomic, monometallic transition metal molecules with nonrelativistic theoretical best estimate (TBE) values computed in the gas phase, making comparison with other nonrelativistic quantum chemistry methods more straightforward than (still useful) back-corrected experimental values.^96^ Herein, I evaluate the performance of various correlated wave function theoretic approaches on the singlet/triplet gaps of the 7 molecule subset in Quest #8 that has a singlet ground state.

MAEs and maximum errors for the Quest #8 singlet/triplet gaps are shown in Figure 5. None of the methods that truncate at double substitutions are quantitatively accurate relative to the TBE benchmarks, but the inclusion of perturbative triple excitations in CCSD­(T) clearly has a large effect on the accuracy of the predicted gaps, reducing the MAE from 0.3 eV with CCSD to 0.07 eV with the inclusion of triples. As one might expect, LinCCD performs similarly well, albeit with a larger maximum error of 1.2 eV. Interestingly, while LinLCCD features a slightly larger MAE than LinCCD at 0.5 eV, it has a smaller maximum error than both LinCCD and CCSD at just 0.7 eV. Of all of the linearized CC approaches, LinLCCD­(hh) performs the worst with a MAE of 0.6 eV, but it still outperforms MP2. As the linearized ladder CCD approaches can be conceptualized as intermediate theories between MP2 and LinCCD, it makes sense that their MAEs fall in a hierarchical order MP2 > LinLCCD­(hh) > LinLCCD > LinCCD. While the results presented here are in line with expectations and are reasonably accurate, they once again point toward a need for incorporating more dynamical correlation into the LinLCCD approximation. Such studies are currently underway in my group.

**Figure 5:**
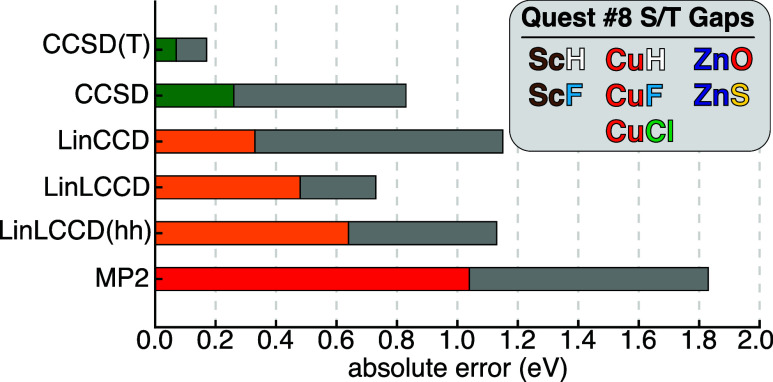
Mean absolute errors (shown as colored bars) and maximum errors (shown as gray bars) for the lowest energy singlet/triplet gaps in Quest #8 as computed by various ΔCC and ΔMP2 methods. The bars are colored according to the category of approximation being applied, where Møller–Plesset perturbation theory is red, linearized CC methods are orange, and nonlinear CC approaches are green. The systems in question are shown in the inset.

### 3.4 Photolysis of Volatile Organic Compounds

The previous results focus mainly on energy differences between the electronic ground state and a high-spin triplet state of the system. This is one example of a ΔCC calculation, where the energy of each electronic configuration is optimized at the self-consistent field level and used as the reference for a subsequent non-Aufbau CC calculation. Excitation energies are then obtained by taking the difference between the CC energies of each respective calculation. In this section, I will explore the calculation of excited state bond dissociation curves via the ΔCC approach to assess LinLCCD for its recovery of potential surfaces of open-shell systems.

Volatile organic compounds are of intense interest in the atmospheric chemistry community and understanding their photochemistry can inform on human health^97,98^ and global climate modeling.^99–101^ One downstream product of CHCl_2_F, a recently phased-out refrigerant, is CF_3_COCl, which is known to decompose under UV irradiation.^102^ The results in Figure 6 show ΔLinLCCD calculations on several of the lowest-energy excited states in CF_3_COCl along the C–Cl bond stretching coordinate. Typically I would include single substitutions within the CC *ansatz* to model open-shell systems, but the presence of singles clusters threatens the stability of the excited-state configuration. By Thouless’ theorem,^103^ single substitutions are equivalent to orbital rotations that could push the desired excited-state solutions toward the ground state. Therefore, a caveat to bear in mind in the following analysis is that the reference determinant for each ΔLinLCCD excited state is spin contaminated and without single substitutions to aid in spin purification the calculated excitation energies are likely underestimated.^104–106^ While approximate spin projection could be employed,^107^ I do not expect this to impact the qualitative validity of the results—especially for the purposes of modeling the topography of each potential surface at large C–Cl distances.

**Figure 6:**
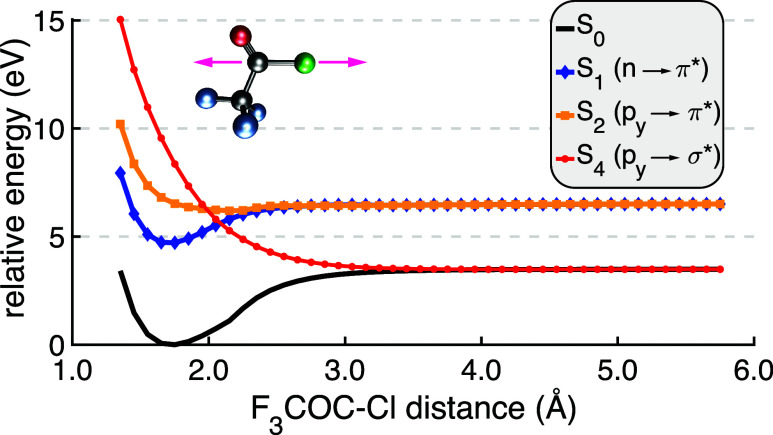
Dissociation of CF_3_COCl along the C–Cl bond using ΔLinLCCD from reference configurations that represent the ground electronic state as well as *n* → π*, *p* → π*, and *p* → σ* transitions.

The dissociation curves for various states of CF_3_COCl are shown in Figure 6. All of the LinLCCD results are qualitatively consistent with the multireference calculations of ref 102. Namely, the *S*_1_ state corresponds to a bound *n* → π* transition at 4.7 eV, which is in good agreement with the experimental band maximum of about 4.9 eV.^102^ Furthermore, the *S*_2_ and *S*_4_ states are unbound and correspond to *p* → π* and *p* → σ* transitions, respectively. Both states lead to free dissociation to two different limits. This is expected, because dissociation along the ground state potential surface should lead to the homolytic cleavage of the C–Cl σ bond, populating the σ* orbital and resulting in the same dissociation limit as the *S*_4_ state. In the case of the *S*_2_ state, the occupied π* orbital corresponds to a qualitatively different configuration at dissociation.

The dissociation limits for the ground state and *S*_2_ states are 3.5 and 6.5 eV, respectively. The former is very close to the extended multistate complete active space second-order perturbation theory (XMS-CASPT2)^108^ dissociation energy for *S*_0_ predicted in ref 102. of 3.44 eV. The energy difference between *S*_0_ and *S*_2_ states at dissociation is 3 eV and is also in excellent agreement with XMS-CASPT2 results. Despite spin contamination, the qualitative curvature of each surface is reasonable and LinLCCD with open-shell singlet references provides excellent bond dissociation energies in the case of CF_3_COCl.

## 4 Conclusions

In summary, I have introduced linearized ladder CCD equations alongside a linear hole–hole ladder approximation and examined their applicability to a range of chemistry contexts. LinLCCD and LinLCCD­(hh) are both robust when static correlation becomes important, and LinLCCD­(hh) serves as an affordable $O\left(n_{o}^{4}n_{v}^{2}\right)$ approximation that can be applied to large systems. I have also shown that the most problematic terms in LinCCD that lead to near-singular correlation energies are the ring and crossed-ring contractions—particularly the exchange contributions therein. It is especially notable that LinLCCD is a size-consistent CID method that is obtained by removing linear terms rather than adding quadratic ones.^33^ With future adaptations of LinLCCD and LinLCCD­(hh) to incorporate more dynamical correlation, these approaches could prove to be quite useful in the design of new computational methodologies for modeling strongly correlated systems reasonably well within single-reference approximations.

## 5. Computational Details

All calculations make use of the resolution-of-the-identity approximation and were performed using a developer version of Q-Chem v6.2.^109^ The A24 and S22 complete basis set limit results were obtained using two-point aug-cc-pVDZ/aug-cc-pVTZ extrapolation using β = 2.51 and α = 4.3 as per Neese and Valeev.^110^ The S22 calculations made use of frozen natural orbitals (FNOs), retaining 99.6% of the natural orbital occupation.^111–113^ The use of FNOs has been shown to be a robust approximation that provides benchmark-quality noncovalent interaction energies on the S22 set.^114^ The L7 calculations were extrapolated to the complete basis set limit using the somewhat smaller Def2-ma-SVP and Def2-ma-TZVP basis sets for heavy atoms and the corresponding Def2-SVP/Def2-TZVP for H atoms.^115–117^ As Neese and Valeev do not provide parameters to minimize extrapolation errors for Karlsruhe basis sets with diffuse functions,^110^ I computed the Hartree–Fock energies for L7 with the Def2-ma-QZVPP basis set and extrapolated the correlation energy with the more typical β = 3 parameter. The L7 calculations are the only ones that use the frozen core approximation. The Quest#8 calculations employ the aug-cc-pVTZ basis for even-handed comparison with the aug-cc-pVTZ reference data in ref 95. (due to limitations in Q-Chem, all *I* angular-momentum functions were removed from the auxiliary basis). Quest#8 systems of triplet multiplicity and the CF_3_COCl potential energy surfaces were calculated using unrestricted Hartree–Fock reference orbitals. Non-Aufbau reference configurations for CF_3_COCl were stabilized using a combination of state-targeted energy projection and initial maximum overlap method algorithms.^118–120^

## Supplementary Material





## Notes

All A24 and S22 data, finite-basis set L7 results, and Quest#8 data (XLSX)
Additional analysis of interaction subtypes in the S22 data set (PDF)
