# Supplementary material for: Diagrammatic Simplification of Linearized Coupled Cluster Theory
Source: J Phys Chem A. 2025 Jun 26;129(31):7251–60. doi: 10.1021/acs.jpca.5c03203 (PMC12337151; doi:10.1021/acs.jpca.5c03203)
Supplement: Supplementary file 2 [file jp5c03203_si_002.pdf]

Supporting Information for
"Diagrammatic Simplification of Linearized Coupled Cluster Theory"

Kevin Carter-Fenk*
Department of Chemistry, University of Pittsburgh,
Pittsburgh, Pennsylvania 15218, USA
kay.carter-fenk@pitt.edu
(Dated: June 18, 2025)

S22 Decomposition of Interaction Types

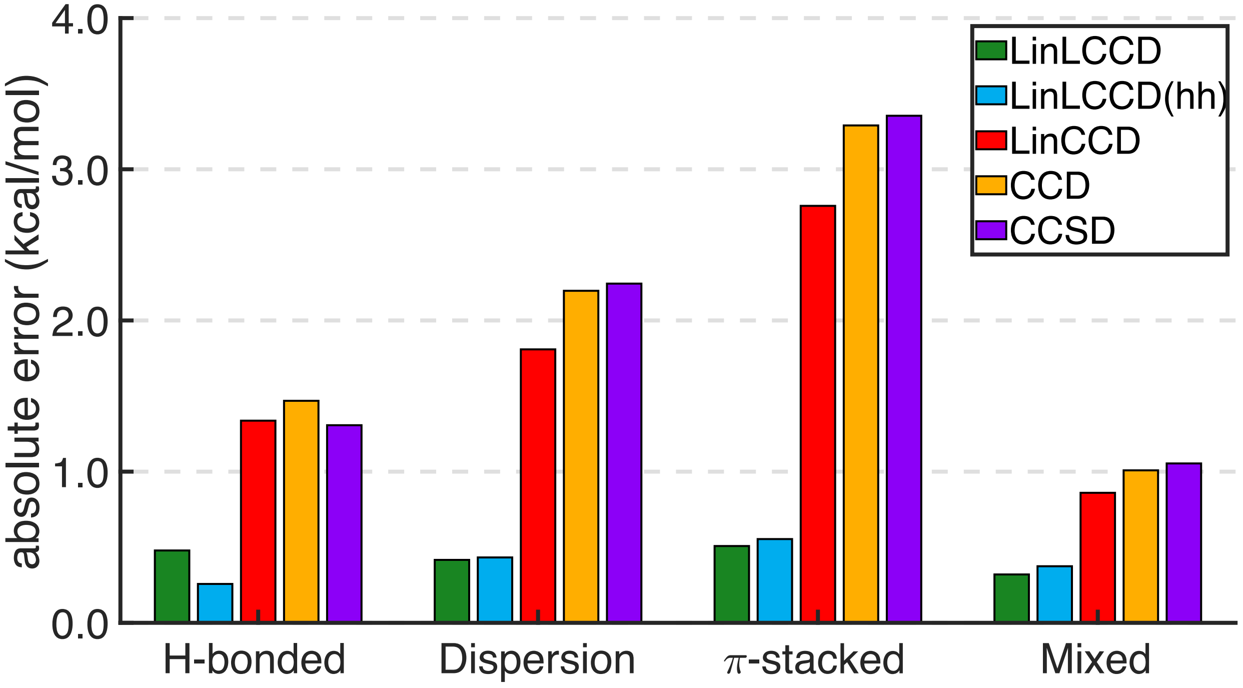

Figure S1. Mean absolute errors across various non-covalent interaction types in the S22 data set.
